# Electrical analysis of c-Si/CGSe monolithic tandem solar cells by using a cell-selective light absorption scheme

**DOI:** 10.1038/s41598-017-15998-y

**Published:** 2017-11-16

**Authors:** Ah Reum Jeong, Sung Bin Choi, Won Mok Kim, Jong-Keuk Park, Jihye Choi, Inho Kim, Jeung-hyun Jeong

**Affiliations:** 1 Korea Institute of Science and Technology (KIST), Seoul, 02792 Republic of Korea; 2 Division of Nano & Information Technology, KIST School, Korea University of Science and Technology, Seoul, 02792 Republic of Korea

## Abstract

A monolithic tandem solar cell consisting of crystalline Si (c-Si)/indium tin oxide (ITO)/CuGaSe_2_ (CGSe) was demonstrated by stacking a CGSe solar cell on a c-Si/ITO solar cell to obtain a photovoltaic conversion efficiency of about 10%. Electrical analyses based on cell-selective light absorption were applied to individually characterize the photovoltaic performances of the top and bottom subcells. Illumination at a frequency that could be absorbed only by a targeted top or bottom subcell permitted measurement of the open-circuit voltage of the target subcell and the shunt resistance of the non-target subcell. The cell parameters measured from each subcell were very similar to those of the corresponding single cell, confirming the validity of the suggested method. In addition, separating the light absorption intensities at the top and bottom subcells made us measure the bias-dependent photocurrent for each subcell. The series resistance of a c-Si/ITO/CGSe cell subjected to bottom-cell limiting conditions was slightly large, implying that the tunnel junction was a little resistive or slightly beyond ohmic. This analysis demonstrated that aside from producing a slightly resistive tunnel junction, our fabrication processes were successful in monolithically integrating a CGSe cell onto a c-Si/ITO cell without degrading the performances of both cells.

## introduction

Multi-junction solar cells (or tandem solar cells) designed with the aim of achieving high photovoltaic conversion efficiencies (PCEs) offer a key technology for reducing the levelized costs associated with photovoltaic energy generation^1^. The single junction limit may be exceeded by harnessing high-energy photons from the sun at higher voltages, which reduces the thermalization loss^2^. Representative multi-junction solar cells are based on group III–V semiconducting materials, which offer a wide range of band gaps and a high crystal quality. These cells have achieved uniquely high efficiencies close to 40%;^3^ however, the high efficiency is offset by penalties in terms of high manufacturing complexity and price (several hundred times higher than the costs associated with commercially-available single-junction solar cells, *i.e*. crystalline Si solar cells). Instead, hybrid tandem solar cells consisting of a wide band gap (E_g_) thin film solar cell and a crystalline Si (c-Si) solar cell have attracted significant interest^4–11^. Such cells offer a pathway to achieving the milestone goal of improving the efficiency beyond 30% while maintaining low fabrication costs^6^. This approach tends to involve, as a bottom subcell, a c-Si solar cell with a sandwich cell architecture (electrode/Si/electrode) that is more compatible with the subsequent deposition of the thin films used to create the top subcell. The sandwich cell architecture is a c-Si PV market-dominating technology with good cost competitiveness. Thus, more beneficially, this approach can utilize the existing manufacturing infrastructure of the c-Si PV industry, for example if the deposition equipment needed to fabricate the top subcell is added.

Perovskite and Cu(In,Ga)(Se,S)_2_ (CIGS) have been considered as promising candidate materials for wide bandgap thin film solar cells, because they offer high band gap tunability and a high PCE exceeding 22%^12,13^. A high PCE and a wide band gap of around 1.7 eV are mandatory for the top-subcell^6^.

With the recent dramatic improvements in perovskite’s performance (its current record efficiency is 22.3%)^12^, many groups have studied perovskite as a top subcell in c-Si/perovskite tandem solar cells^4,5,8,9^, leading to the achievement of a peak efficiency of 23.6% in a two-terminal monolithic integrated structure^10^ and a peak efficiency of 26.4% in a 4-terminal mechanically stacked structure^14^. However, perovskite solar cells contain a significant amount of lead (Pb), which is harmful to the human body and shows very poor long-term stability upon exposure to moisture, light, bias, and heat^15,16^. The issues need to be addressed for the successful commercialization of these cells.

CIGS thin film solar cells display excellent long-term stability, and can be fabricated using minimal or no toxic elements. For example, Cd can be removed by replacing the CdS buffer with a Cd-free buffer, such as Zn(O,S) and ZnSnO. However, the CIGS solar cell provides its highest efficiency at a narrow band gap of around 1.15 eV, which is too low for use in top subcells. A wider band gap absorber, such as CuGaSe_2_ (CGSe), which has a band gap of 1.68 eV, displays at best a relatively low PCE of 11.2%^17^. This PCE is too low for use in top subcells. The PCE of the top subcell must exceed 14% to obtain a tandem cell PCE above 25% and must exceed 18% to obtain a tandem cell PCE above 30%^6,18^. The primary factor underlying the low PCE of the CGSe solar cell is a high V_OC_ deficit (=E_g_/q − V_OC_), attributed to the high recombination rate resulting from either a high density of bulk and/or surface defects^19,20^ or inappropriate alignment of the conduction band minima (CBO) of the CdS buffer and CGSe absorber^21^. Another problem is that c-Si and CGSe solar cells do not have an optimal tunnel junction to enable easy transport of the carriers between the top and bottom subcells. The only approach currently available involves the use of a transparent conducting oxide (TCO) layer, such as an indium tin oxide (ITO) film, as an interlayer between the CGSe film and the c-Si emitter; however, a highly resistive gallium oxide (GaO_x_) layer tends to form at the interface between ITO and CIGSe during deposition, producing a counter diode that blocks carrier transport^22,23^. For this reason, the highest PCE achieved to date using a tandem structure was only 5.1%^11^.

**Figure 1:**
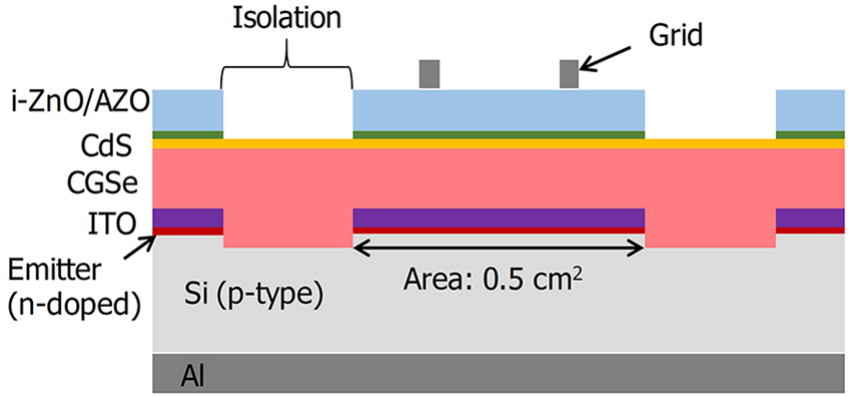
Diagram showing a cross-sectional view of the fabricated c-Si/ITO/CGSe monolithic tandem solar cell.

Despite the issues described above, it is very valuable to find methods for improving the performances of c-Si/TCO/CGSe monolithic tandem cells. Such methods include searching for novel ways to reduce the high V_OC_ deficit of the CGSe solar cell or creating more ohmic junctions between c-Si and CGSe. In any effort designed to overcome these critical issues, analysis of the root cause underlying the performance degradation in tandem cells is mandatory. It is particularly difficult in two-terminal monolithic-integrated tandem cells to identify the causes underlying their subcell degradation. Because subcells within the structure are connected in series and cannot be separated, identifying the damaged subcell associated with cell performance degradation during fabrication or characterization tends to be difficult. A lack of methods for characterizing the performances of subcells increases the difficulties associated with successfully fabricating tandem cells. Few studies have addressed the effects of the subcell breakdown voltage and shunt loss on the electrical properties of tandem cells^24,25^. In this study, we individually characterized the electrical performances of the top and bottom subcells in a monolithic c-Si/ITO/CGSe tandem solar cell developed in our laboratory; the cell structure is shown in Fig. 1.

## Results and Discussion

### Method for individually characterizing the performance of the top subcell or bottom subcell

**Figure 2:**
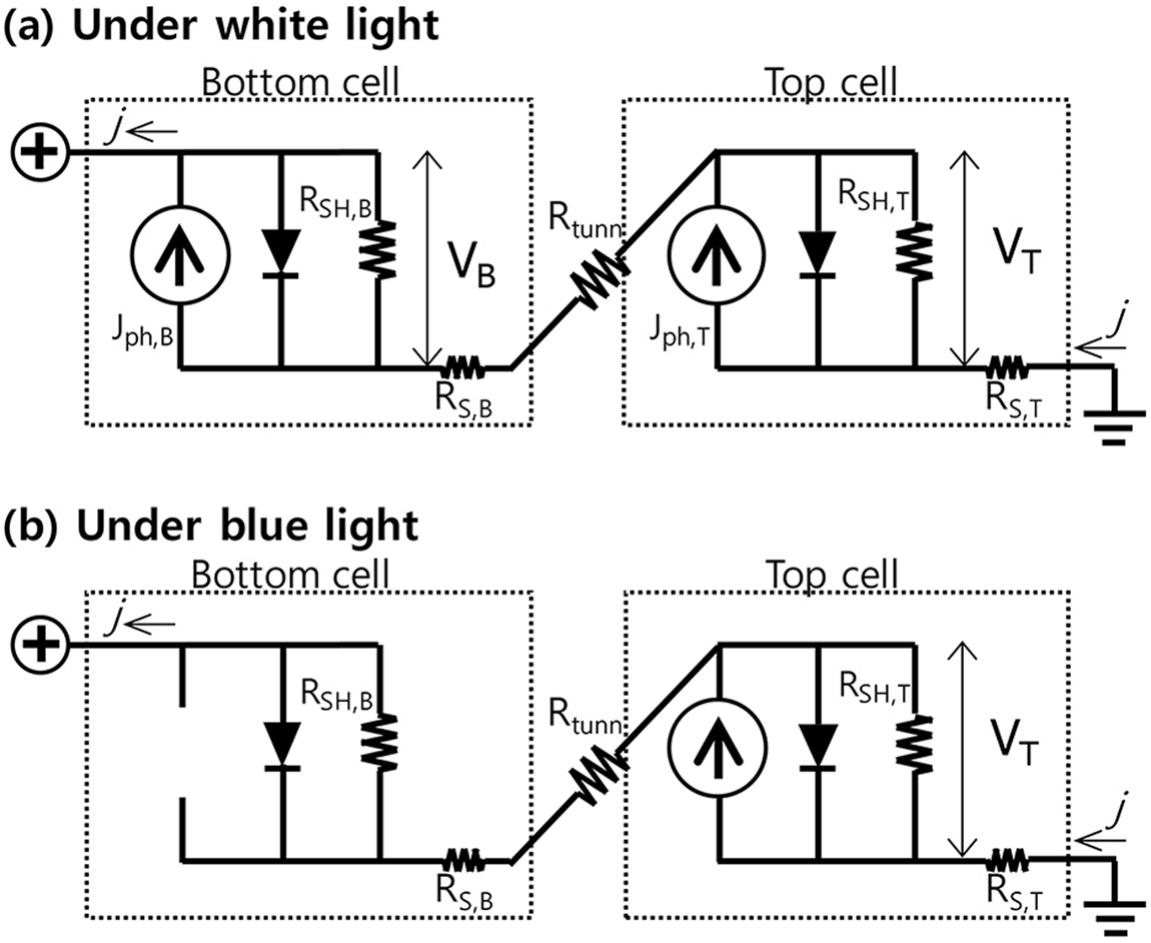
Electrical circuit of a tandem cell subjected (**a**) to white light illumination and (**b**) blue light illumination. J_ph_, R_SH_, and R_S_ indicate the photocurrent, shunt resistance, series resistance, respectively. V indicates the voltage across the top or bottom subcell. Here, the subscripts B and T represent the bottom and top subcells, respectively.

Figure 2 illustrates the electrical circuit of a tandem solar cell in which two subcells were connected in series. Here, all lights entered the top subcell first. Illumination of the cell with white light across the full solar spectrum generated a photocurrent and photovoltage in both the top and bottom subcells (see Fig. 2(a)). Because the top and bottom subcells were connected in series in the tandem cell, the voltage across the tandem cell (V_TOT_) was determined by the summation of each subcell’s voltage (V_T_ + V_B_), and the current was determined by the lowest current in the subcells. Under white light illumination, the performance of each subcell could not be determined from the *j-V* characteristics of the tandem cell alone unless the properties of a single junction cell corresponding to each subcell were determined separately.

Unlike the case presented in Fig. 2(a), the absorption of light in either the top or bottom subcell generated a photovoltage and photocurrent only in the subcell in which light was absorbed. As shown in Fig. 2(b), blue light illumination (at an energy higher than the absorber band gap in the top subcell) did not generate a photocurrent in the bottom subcell. In this case, the open-circuit or short-circuit conditions could be defined as follows.

Under open-circuit conditions, the current in the tandem cell was zero, and a voltage due to the light-induced current formed across the top subcell. The magnitude of the voltage depended slightly on the magnitude of the absorbed light but was almost equal to the open-circuit voltage generated in the top subcell alone (V_OC,T_) under one-sun illumination. The open-circuit voltage of a well-functioning solar cell is a logarithmic function of the photocurrent and is not sensitive to small variations in the light intensity^26^. Because light was not absorbed in the bottom subcell, the voltage across the bottom subcell was zero. Consequently, under open-circuit conditions, the total voltage across the tandem cell (V_T_ + V_B_) was expected to be identical to the V_OC,T_. In other words, the open-circuit voltage of the top subcell (V_OC,T_) could be obtained from the x-axis intercept of the *j-V* curve of the tandem cell measured under blue light illumination. On the other hand, consider a case in which the top subcell was partially shunted. Applying the principles described above, the open-circuit voltage of the top subcell as reduced by a leakage current through the shunt resistance could be extracted from the measured blue light *j-V* curve of the tandem cell.

Under short-circuit conditions, the total voltage should be zero, and V_B_ = −V_T_ if the series resistance of each cell was negligible. Under blue light illumination, the photocurrent was not generated in the bottom subcell. Therefore, the bottom subcell had no way to allow for the passthrough of the top subcell photocurrent except via paths such as the shunt resistance and e-h recombination in the diode under a reverse bias. If the shunt resistance of the bottom subcell was sufficiently high (R_SH,B_ ≫ 1), the current passing through the bottom subcell was negligible compared to the photocurrent of the top subcell. Consequently, the top subcell could be approximated as operating under open-circuit conditions, *i.e*., V_T_ = V_OC,T_ in the case R_SH,B_ ≫ 1. On the other hand, if the bottom subcell were partially shunted (R_SH,B_ ≪ R_SH,T_), a portion of the top subcell photocurrent could flow through the bottom subcell (which reduced the recombination current within the top subcell), thus slightly decreasing V_T_. Nonetheless, V_T_ was similar to V_OC,T_, (V_T_ ~ V_OC,T_) because the voltage was less sensitive to the current variations, as stated above. Here, the voltage across the shunt resistance of the bottom subcell was the same as V_B_ (= −V_T_ ~ −V_OC,T_). Consequently, the short-circuit current (j_SC_) of the tandem cell should pass through the shunt resistance of the bottom subcell under a bias of V_B_ (~ −V_OC,T_), assuming that the reverse diode recombination current of the bottom subcell was negligible. These conditions lead to the relation R_SH,B_ = V_OC,T_/j_SC_.

Red light illumination (with an energy lower than the absorber band gap of the top subcell) resulted in absorption only in the bottom subcell, which generated a photovoltage and photocurrent only in the bottom subcell. Thus, the open-circuit voltage of the bottom subcell (V_OC,B_) and the shunt resistance of the top subcell (R_SH,T_) could be extracted according to the approach described above under blue light illumination. In summary, inducing selective light absorption by either the top or bottom subcell enabled characterization of the individual electrical properties of the constituent subcells within a monolithic tandem solar cell.

**Figure 3:**
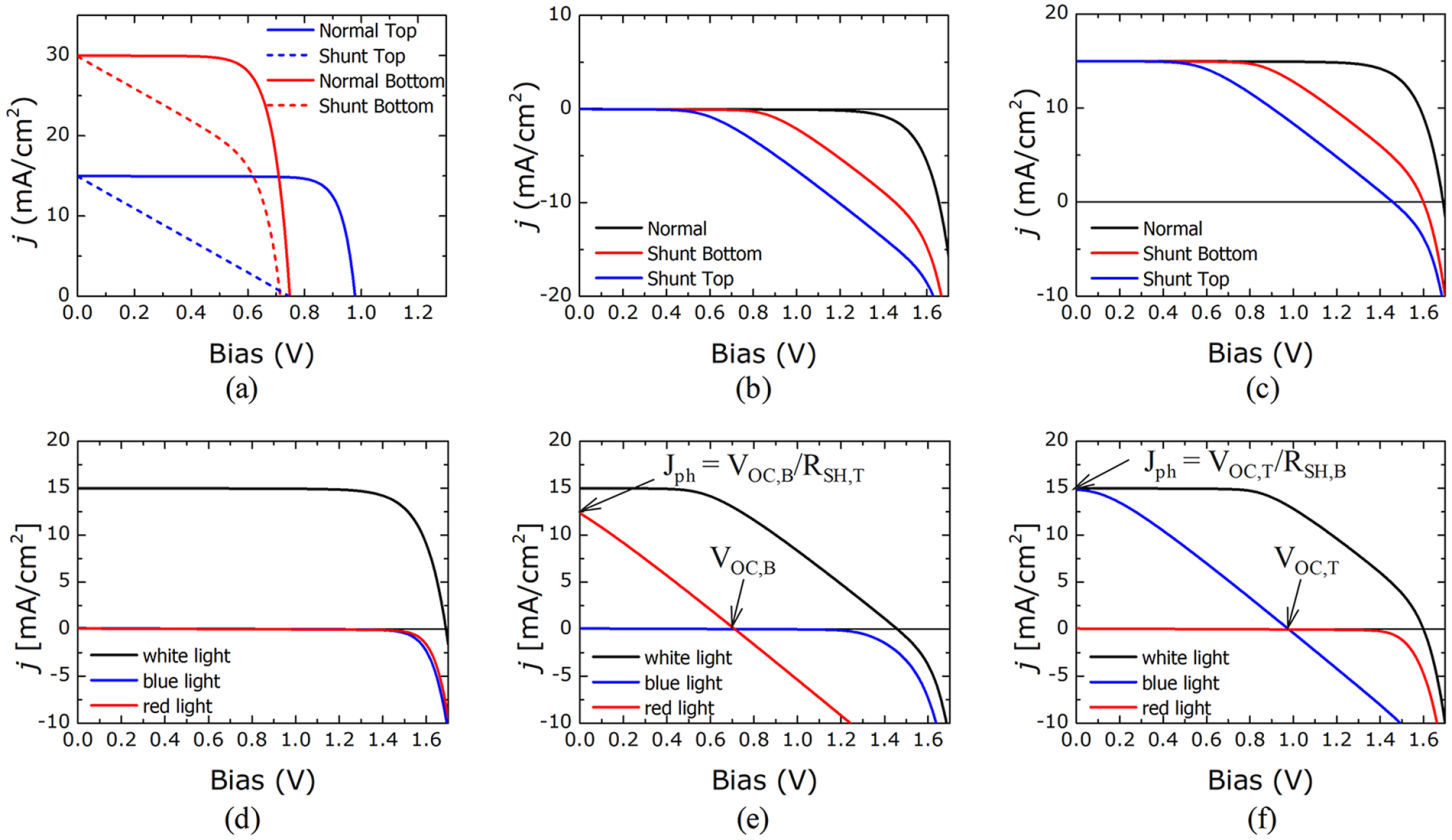
(**a**) *j-V* characteristics of standalone top and bottom cells under well-functioning or shunt conditions, respectively. (**b**,**c**) *j-V* characteristics of a tandem cell in which the subcells are all well-functioning or partially shunted (**b**) under the dark state, or (**c**) under white light illumination. (**d**–**f**) *j-V* characteristics under white light, blue light, or red light illumination of a tandem cell in which (**d**) the subcells are all well-functioning, (**e**) the top subcell is shunted, or (**f**) the bottom subcell is shunted.

Figure 3 plots the PSPICE-simulated *j-V* characteristics of the tandem cell for the case of a well-functioning or shunted subcell (see Fig. 3(a)) under various illumination conditions: dark, white light, blue light, or red light. Figure 3(b) and (c) compare the dark *j-V* and white light *j-V* characteristics of tandem cells in well-functioning or partially shunted states. The dark *j-V* (Fig. 3(b)) and white light *j-V* (Fig. 3(c)) curves could not distinguish which subcell was shunted unless the individual characteristics of each subcell were known. By contrast, Fig. 3(d–f) show that the blue light or red light *j-V* measurements indicated exactly which subcell was shunted. First, consider the case in which both subcells functioned well (i.e., were not shunted), as shown in Fig. 3(d). Blue light or red light illumination could not induce a substantial short-circuit current because a photocurrent was not generated in one of the subcells: A short-circuit current is limited by the smallest current in either of the top and bottom subcells. Second, consider the case in which the top subcell was shunted, as shown in Fig. 3(e). The photocurrent generated in the bottom subcell by red light absorption could pass easily through the shunt path of the top subcell, whereas the photocurrent due to blue light absorption could not pass through the bottom subcell. Third, consider the case in which the bottom subcell was shunted, as shown in Fig. 3(f). Unlike in the second case, the photocurrent generated in the top subcell by blue light absorption could pass through the shunt path of the bottom subcell, whereas photocurrent due to red light absorption could not pass through the top subcell.

As indicated in Fig. 3(e) and (f), the open-circuit voltages of the top subcell could be evaluated from the x-intercept of the blue light *j-V* curves, and those of the bottom subcell could be evaluated from the x-intercept of the red light *j-V* curves.

### Application to the c-Si/ITO/CGSe tandem cell: CASE 1 – well-functioning cell

**Figure 4:**
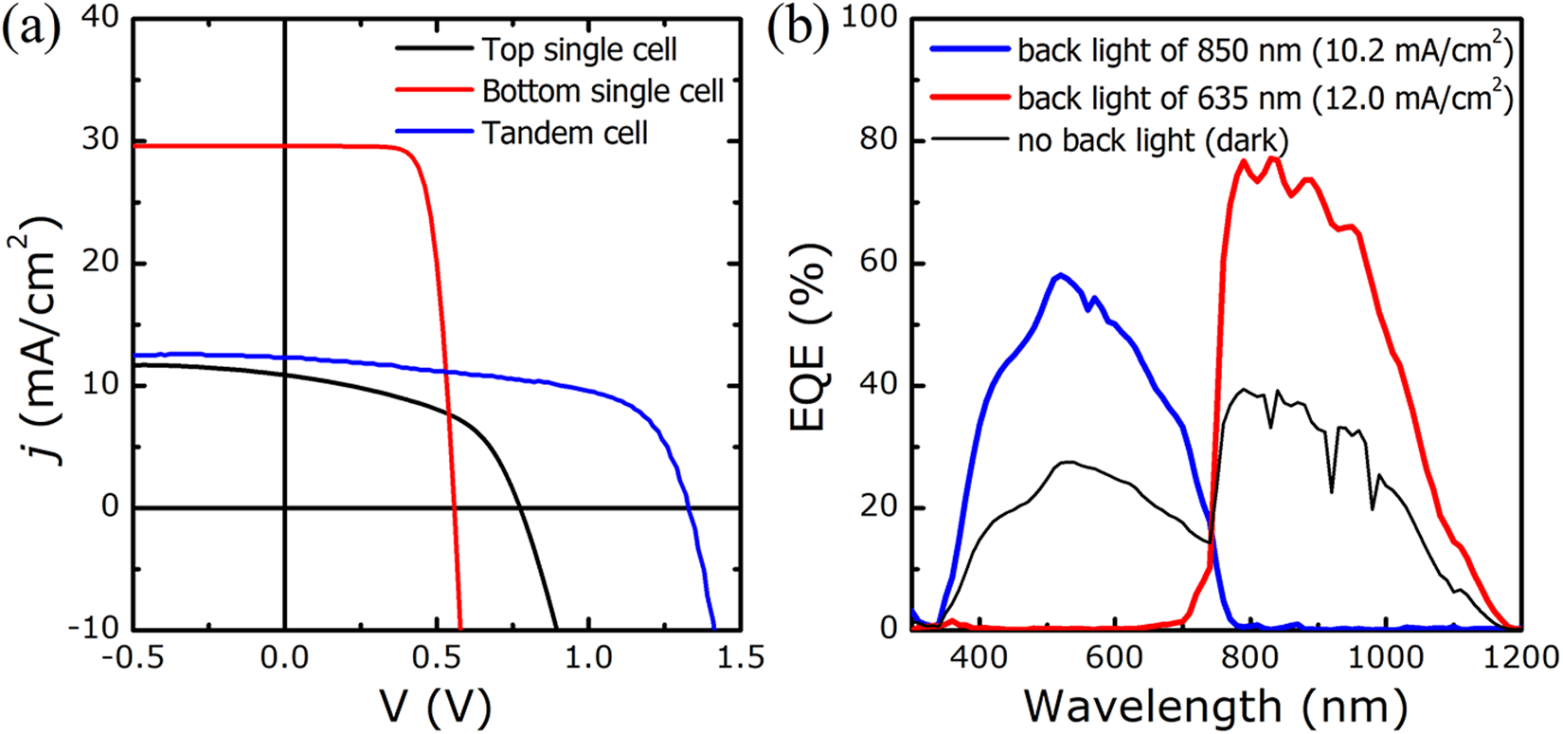
Photovoltaic performances of the Si/ITO/CGSe tandem solar cell. (**a**) *j-V* characteristics (compared with those obtained from the single subcells). (**b**) External quantum efficiency spectra obtained with no back light illumination or with 850 nm or 635 nm back light illumination.

The method described in the previous section was applied to a c-Si/ITO/CGSe monolithic tandem cell fabricated in our laboratory (see the experimental section). Figure 4(a) compares the *j-V* characteristics of the c-Si/ITO/CGSe tandem solar cell with those of the single CGSe solar cell and c-Si solar cell. Here, the single CGSe solar cell was fabricated on an ITO-coated Si wafer, rather than on soda-lime glass. The efficiency of the tandem cell was 9.7% (V_OC_: 1.328 V, J_SC_: 12.3 mA/cm^2^, FF: 59.4%), which to our knowledge is the highest value yet reported. (The highest PCE reported previously for a similar cell structure was 5.1%^11^) The efficiencies of the single cells were 4.2% for the ITO/CGSe cell (V_OC_: 0.774 V, J_SC_: 10.9 mA/cm^2^, FF: 49.4%), and 12.3% for the c-Si/ITO cell (V_OC_: 0.558 V, J_SC_: 29.6 mA/cm^2^, FF: 74.2%).

The open-circuit voltage of the tandem cell was the same as the sum of the values obtained from the single c-Si and CGSe cells. Considering that the top and bottom subcells were connected in series, no V_OC_ loss occurred for the monolithically stacked CGSe solar cell on top of the c-Si solar cell. The fill factor of the tandem cell was improved relative to the value obtained from the single CGSe cell but degraded relative to the value obtained from the single c-Si cell. The mechanism by which the V_OC_ and FF were achieved in the tandem cell will be discussed below, along with the results obtained from the electrical analyses described here.

The short-circuit current of the tandem cell was slightly larger than that of a single CGSe cell and significantly smaller than that of a single c-Si cell. It was difficult to obtain a mechanistic description of photocurrent generation and collection in the tandem cell without independent knowledge of the optical and electrical losses in the top and bottom subcells. The quantum efficiency (QE) spectrum suggested some insights into how the photocurrent in the tandem cell was achieved. The QE is obtained by measuring the photocurrent at a single photon energy, and photocurrents could only be produced in the bottom and top subcells simultaneously for photon energies that could be absorbed in both subcells. Therefore, it would be impossible to measure the exact photocurrent generated by the subcells at each photon energy without modifying the illumination configuration. Back light illumination was applied during the external quantum efficiency (EQE) measurements: 850 nm monochromatic light was used to characterize the top subcell, and 635 nm monochromatic light was used to characterize the bottom subcell.

As shown in Fig. 4(b), the CGSe top subcell showed a maximum collection efficiency of 60%, and the c-Si bottom subcell showed a maximum of 80%. The short-circuit current from the top subcell was calculated from its EQE spectrum to be 10.2 mA/cm^2^, similar to that of a single CGSe cell (10.9 mA/cm^2^). This low current was attributed to the fact that the CGSe cell technology used in the fabrication of the tandem cell was not optimal. The low PCE of the CGSe solar cell is a major cause of the relatively low tandem cell efficiency compared with its potential performance. On the other hand, the short-circuit current from the bottom subcell was calculated, from its EQE spectrum, to be 12.0 mA/cm^2^, significantly lower than the single cell value (29.6 mA/cm^2^). This reduction was attributed mostly to light absorption by the top subcell in the short wavelength range (above the band gap energy of the CGSe absorber) and partly to long-wavelength optical losses via reflection and absorption in the AZO, CGSe, and ITO thin films of the top subcell. Maximizing the photocurrent and optimizing the cell structure of the tandem cell requires a systematic optical analysis.

The magnitude of the short-circuit current, 12.3 mA/cm^2^, obtained from the *j-V* measurements in the tandem cell did not match those calculated from the EQE spectra, 10.2 mA/cm^2^ for the top subcell and 12.0 mA/cm^2^ for the bottom subcell. Because the subcells were connected in series, the J_SC_ values were expected to be the lower of the calculated values obtained from the EQE spectra. However, the actual short-circuit current was closer to the higher calculated value. This mismatch in the J_SC_ values obtained from the *j-V* measurement and the EQE spectral calculations could be explained as follows. The photocurrent in the CGSe solar cell exhibited a strong bias dependency, as shown in the *j-V* curve of the single top cell, in Fig. 4(a). When the photocurrents generated in two subcells (connected in series) differ, a negative bias may be applied across the subcell having a lower photocurrent due to the excess photocurrent in the adjacent subcell. A negative bias across the top subcell would improve the current collection efficiency and increase the photocurrent to 12 mA/cm^2^. This bias effect may be negligible in the EQE measurements because the probe and back light illumination intensities were very weak.

**Figure 5:**
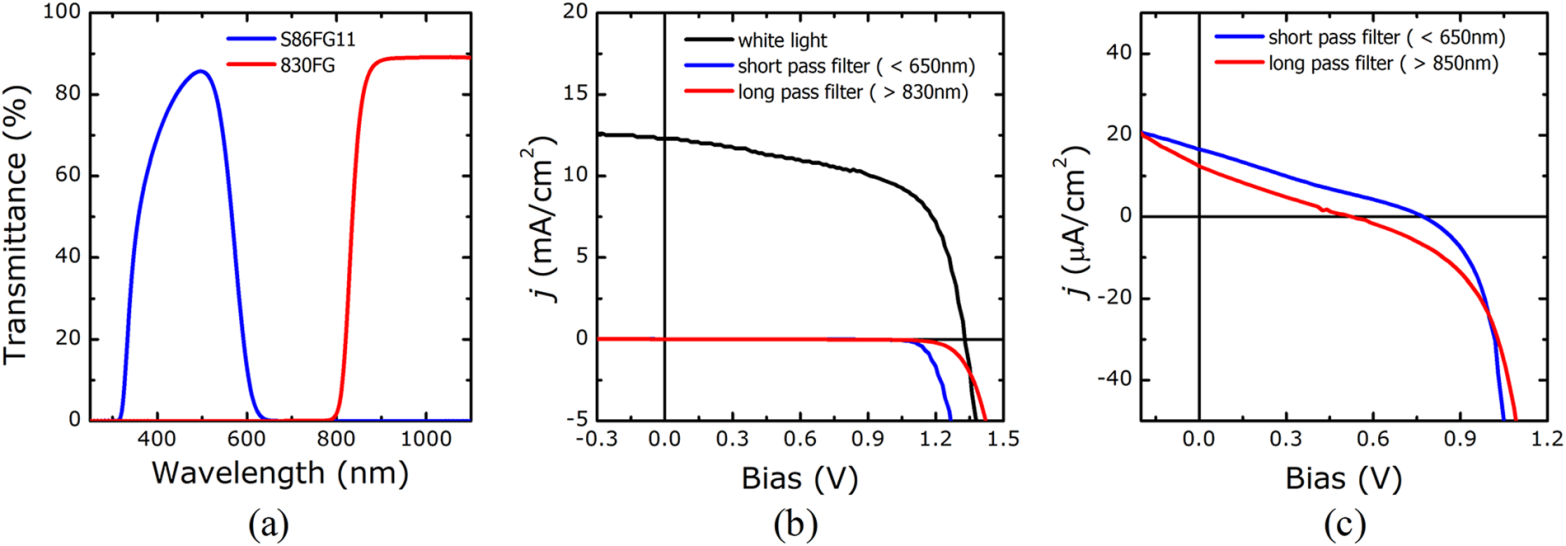
(**a**) Light transmittance spectra of the optical filters used in the measurements. (**b**) Light *j-V* characteristics of the tandem cell measured under white light, blue light (with a filter S86FG11), or red light (with a filter 830FG) illumination. (**c**) *j-V* curves collected under blue light or red light illumination, rescaled from (**b**).

The above discussion of the tandem cell efficiency (Fig. 4) assumed that the performances of a single CGSe cell and a single c-Si cell remained unchanged upon integration in a tandem cell. It was, however, impossible to identify the exact status of each subcell in the tandem cell. The performances of the subcells were examined by measuring the *j-V* characteristics under cell-selective light absorption conditions applied to the tandem cell, as explained in the previous section. In the measurement, a short pass filter was used to permit light transmission at wavelengths below 650 nm, and a long pass filter allowed transmission at wavelengths above 830 nm (see Fig. 5(a)). With the short and long pass optical filters in place, one of the subcells did not produce a photocurrent. Thus, the photocurrent generated in each subcell was mostly suppressed by its adjacent subcell unless the latter was shunted. Figure 5(b) presents the results obtained from exactly this case. The photocurrent disappeared upon application of the optical filters. Rescaling the y-axis of the graph to highlight the smaller current (see Fig. 5(c)) revealed that a very small photocurrent was present, resulting in open-circuit voltages with both a short pass filter and a long pass filter, respectively.

The analysis described in section (1) permitted extraction of V_OC,T_ and R_SH,B_ from the *j-V* curve obtained using the short pass filter (corresponding to the case of blue light illumination), whereas V_OC,B_ and R_SH,T_ were obtained using the long pass filter (corresponding to red light illumination), as summarized in Table 1. These results revealed that both the top and bottom subcells were characterized by sufficiently large shunt resistances. The measured open-circuit voltage of the top subcell was almost the same as that of the single CGSe cell. Together with the previous EQE results, which indicated that the photocurrent of the top subcell was equal to that of the single CGSe cell, these results demonstrated that the CGSe cell performance was preserved in the c-Si bottom cell without loss. The bottom subcell showed a large shunt resistance similar to that of the top subcell, but its open-circuit voltage was slightly smaller than that of the single c-Si cell. The lower open-circuit voltage was attributed to reduced light absorption due to screening by the top subcell. These results demonstrated that the c-Si solar cell was highly tolerant of the elevated temperatures and Se environment imposed during CGSe film growth. These fabrication conditions did not negatively influence the performance of the CGSe subcell.

**Table 1:** The photovoltaic parameters of the subcells in a tandem cell (Fig. 4), obtained using the cell-selective light absorption method.

Constituent cell	V_OC_ (V)	R_SH_ (Ω)
Top cell (CGSe)	0.772	43,750
Bottom cell (c-Si)	0.525	45,411

**Figure 6:**
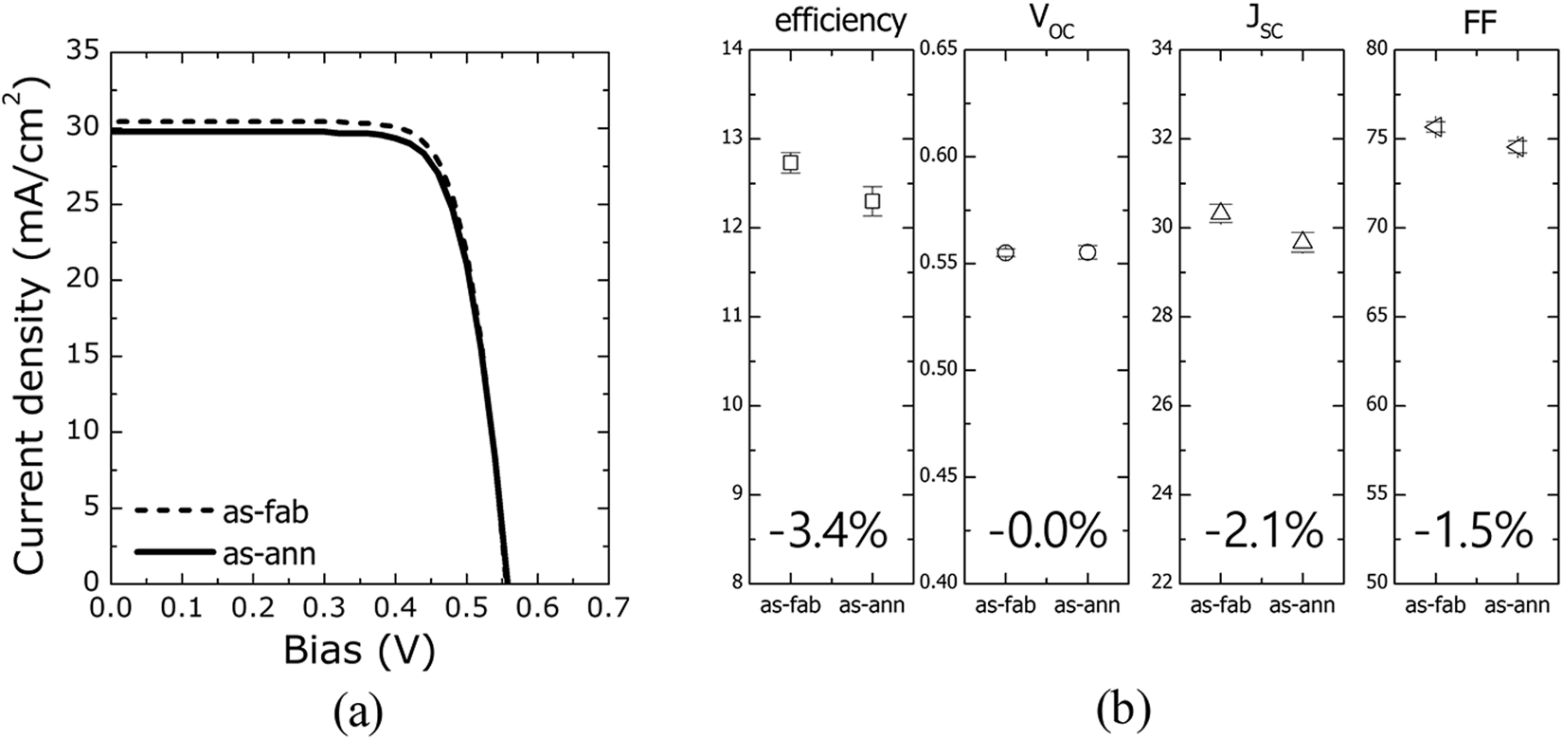
Comparison of the photovoltaic performances of a c-Si solar cell before and after annealing at 550 °C during Se evaporation: (**a**) *j-V* characteristics and (**b**) photovoltaic parameters.

The strong tolerance of the c-Si solar cell to elevated temperatures was revisited by comparing the photovoltaic performances before and after annealing at 550 °C during Se evaporation (conditions similar to the CGSe film growth conditions). As shown in Fig. 6, annealing did not degrade the performance of the c-Si cell, except for a slight reduction in the short-circuit current. The J_SC_ reduction did not appear to arise from cell degradation, but may have been related to uncertainties in the active area estimates or *j-V* measurements.

It is worth highlighting an anomaly observed in Fig. 5(b): long-wavelength light illumination apparently required a higher voltage than was needed under short-wavelength light illumination to obtain an identical current in the tandem cell. This phenomenon may have resulted from a secondary barrier to the current in the CdS/CGSe layers of the top subcell. The secondary barrier was tentatively attributed to the accumulation of negative charges at the interface due to the presence of either deep-level acceptor traps within the CdS layer or Se vacancies related to deep-level defects near the absorber surfaces^27–29^. Blue photon absorption by the CdS buffer generated holes that passivated the deep-level defects in the buffer or the absorber surface and, in turn, reduced the secondary barrier^27^. The voltage drop across the CdS buffer was, therefore, significantly reduced. Blue photons could not be absorbed by the CdS buffer layer in the presence of the long pass filter, and the CdS buffer became more resistive, thereby increasing the diode voltage, as shown in Fig. 5(b).

The framework described above successfully characterized the V_OC_, R_SH_, and J_SC_ values of the individual subcells in a monolithic tandem cell; however, this approach did not reveal the fill factor or the bias-dependence of the photocurrent, which were measured from the single CGSe cell (see Fig. 4(a)). In other words, it is not yet clear whether the CGSe cell photocurrent preserved its strong bias-dependence upon stacking on the c-Si subcell. The quality of the tandem cell tunnel junction employed in this study also raised concerns. We postulated that the quality of the tunnel junction against carrier transport could be determined from the level of the series resistance extracted from a diode analysis performed on the tandem *j-V* characteristics.

**Figure 7:**
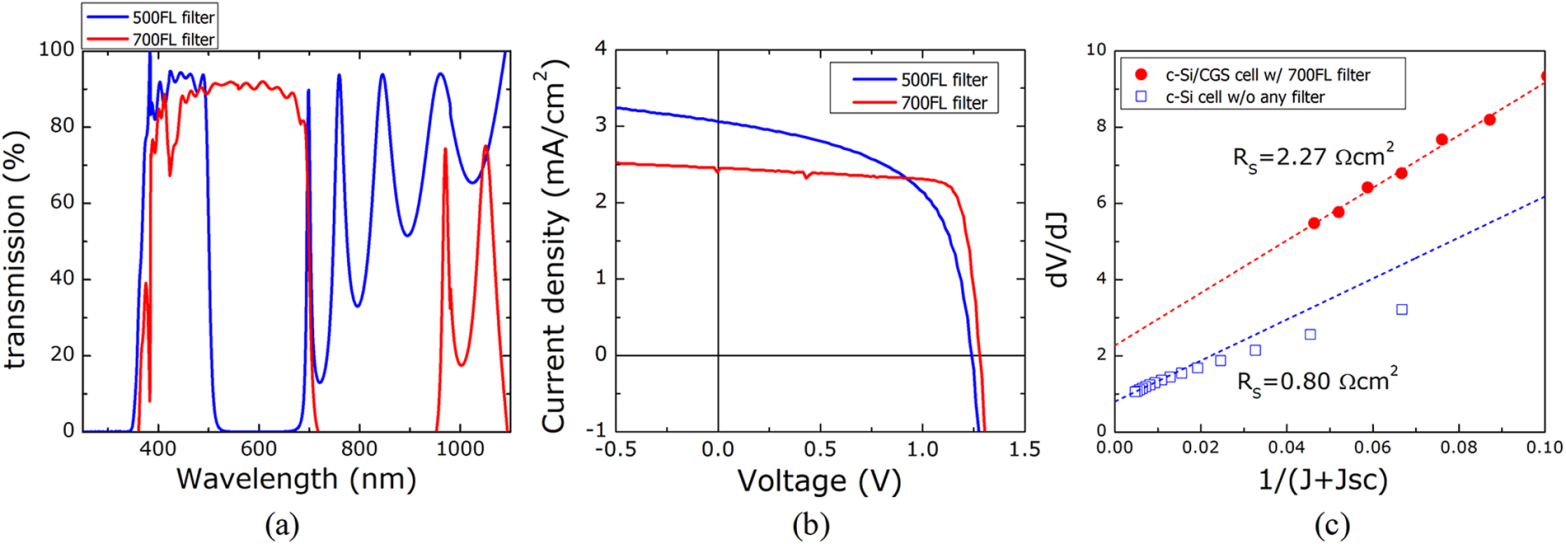
(**a**) The transmission spectra of the optical filters used here. (**b**) Light *j-V* characteristics of the c-Si/ITO/CGSe tandem solar cell in the presence of the 500FL or 700FL filters. (**c**) Results of the diode parameter analysis for calculating the series resistance of the c-Si/ITO/CGSe tandem cell and a single c-Si cell.

We postulated that adjusting the relative amount of light absorbed in the top cell and the bottom cell could be used to characterize the bias-dependent photocurrent properties of the subcells. For this purpose, we used two optical filters with the transmission spectra shown in Fig. 7(a). The 500FL filter transmitted light over a narrow range of short wavelengths and a wide range of long wavelengths, whereas the 700FL filter transmitted light over a relatively wide range of short wavelengths and a relatively narrow range of long wavelengths. The photocurrents in the top subcell (j_QE,T_) and the bottom subcell (j_QE,B_) under AM1.5 G 1 sun conditions could be calculated from the EQE spectra (see Fig. 4(b)), the transmission spectra of the optical filters in place, and the AM1.5 G solar spectrum. The results revealed that j_QE,T_ = 2.8 mA/cm^2^ and j_QE,B_ = 7.6 mA/cm^2^ for 500FL and j_QE,T_ = 8.1 mA/cm^2^ and j_QE,B_ = 1.1 mA/cm^2^ for 700FL. Therefore, use of the 500FL filter induced a lower photocurrent in the top subcell, which limited the photocurrent of the tandem cell and clearly revealed the *j-V* characteristics of the top subcell. On the other hand, the 700FL filter allowed the bottom subcell to limit the photocurrent of the tandem cell, revealing the *j-V* characteristics of the bottom subcell. Figure 7(b) reveals that the absorption of different light intensities by the two subcells (as described above) allowed the elucidation of the characteristics of the subcells. The use of the 500FL filter revealed that the top subcell preserved the strong bias-dependence of the photocurrent observed in the single CGSe cell (see Fig. 4(a)). Use of the 700FL filter confirmed that the bottom subcell displayed no photocurrent bias dependence, as is typical of a single c-Si cell. Considering that the tandem cell showed a strong bias-dependence of the photocurrent (see Fig. 4(a)), we concluded that the c-Si/ITO/CGSe tandem cell operated under top-subcell limiting conditions.

The quality of the junction layer introduced between the top and bottom subcells was next examined. This layer must permit easy passthrough of carriers between the top subcell and the bottom subcell and form ohmic contacts with the adjacent subcells. A junction that is resistive or insufficiently ohmic can increase the series resistance. The layer quality, therefore, could be assessed by measuring the series resistance in an indirect way through a diode parameter analysis of the tandem *j-V* characteristics. The photocurrent in the tandem cell was limited by the top subcell and, therefore, was strongly dependent on the bias. The bias dependence of the photocurrent might be related to the poor properties of the p-n junction between the CGSe and CdS, for example, a high conduction band barrier, a high interface recombination, or a very low photocarrier diffusion length^30^. These features could render the diode parameter analysis impractical. To avoid interference by the top subcell in the diode parameter analysis (the details of which are described elsewhere^26^), the 700FL filter data was examined so that the photocurrent was limited by the bottom subcell and, thus, was not influenced by the poor p-n junction of the top subcell. The series resistance of the tandem cell was estimated to be 2.27 Ωcm^2^. As shown in Fig. 7(c), this value was much higher than that of the single c-Si cell (0.82 Ωcm^2^), which meant that the junction layer employed in the tandem was slightly resistive or insufficiently ohmic. The problems associated with the junction layer likely arose from a reaction between the ITO and CGSe or from the treatment conditions. The mechanistic details will be examined in a future study.

### Application to the c-Si/ITO/CGSe tandem cell: CASE 2 – partially shunted cell

**Figure 8:**
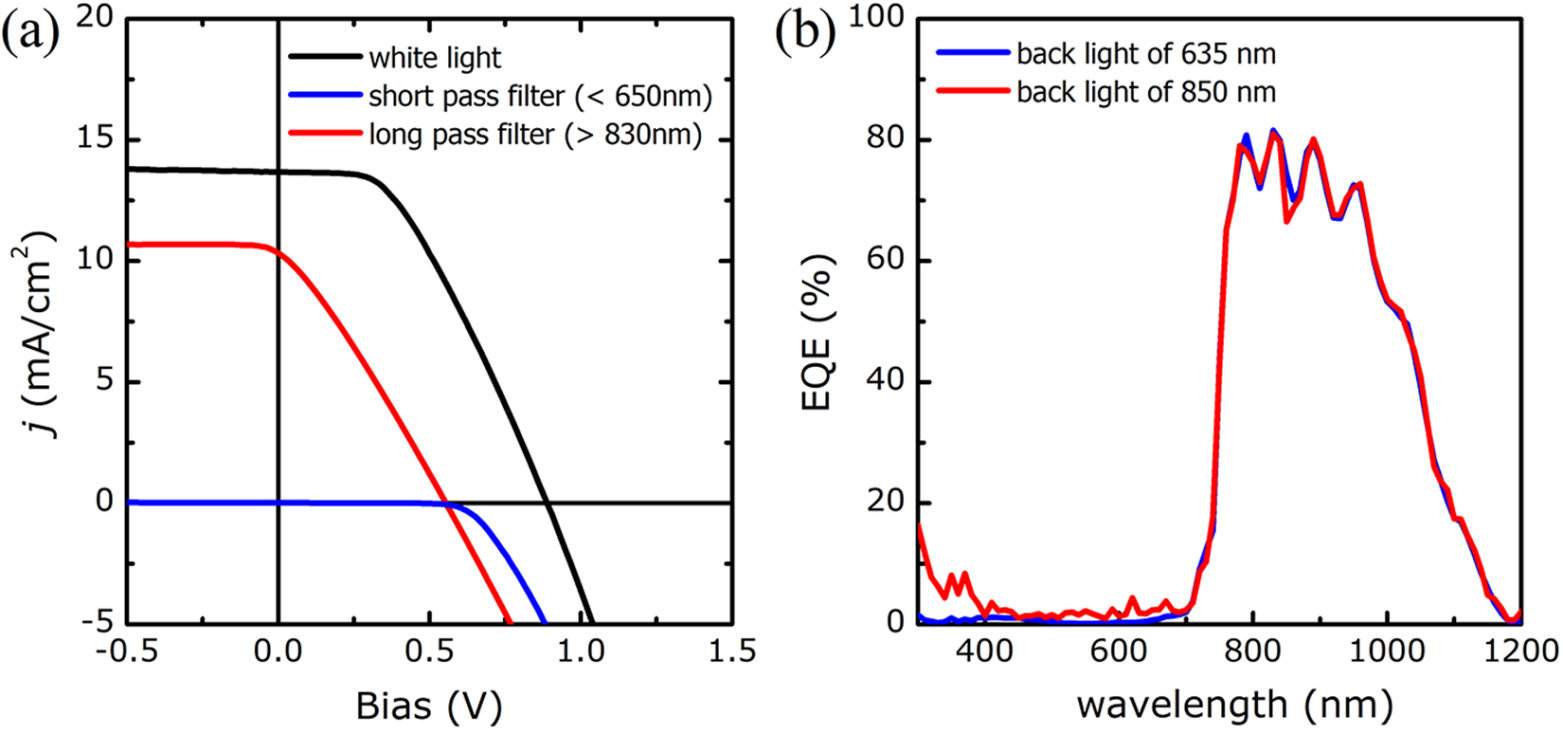
(**a**) *j-V* characteristics of a damaged c-Si/ITO/CGSe tandem solar cell measured under white light, blue light (with a filter S86FG11), or red light (with a filter 830FG) illumination and (**b**) its EQE spectra obtained with 850 nm or 635 nm back light illumination.

To examine an instance of shunt failure in a tandem cell we next conducted an electrical analysis based on cell-selective light absorption on a partially damaged tandem cell. For this analysis we used a cell that had been fabricated using the well-functioning cell fabrication process described in CASE 1, but which had been accidentally damaged by mechanically pressing with a sharp tip during sample handling. The damaged cell examined in this analysis represents an example of the consequences of a shunt failure. In these cells, such failures can have several causes, including the unintended creation of an electrical path due to CGSe delamination or to incomplete pn junctions of the CGSe top subcell. The PCE was severely degraded to 5.2% (V_OC_: 0.888 V, J_SC_: 13.7 mA/cm^2^, FF: 42.6%), much lower than the efficiency of 9.7% obtained in CASE 1. The *j-V* curve measured under white light illumination, as shown in Fig. 8(a), revealed that the tandem cell behaved as if it had a high series resistance, and there was no way to tell which subcell was damaged.

Figure 8(a) also presents the *j-V* characteristics measured under cell-selective light absorption. Application of the short pass filter (transmitting light below 650 nm) induced light absorption only by the CGSe top subcell. These conditions generated a photocurrent in the top subcell; however, the photocurrent of the tandem cell was negligible, indicating that the bottom subcell was not shunted. On the other hand, application of a long pass filter (transmitting light above 850 nm) induced photocurrent generation only in the bottom subcell. The resultant photocurrent was very large compared to the photocurrent measured with top subcell illumination. These results suggested that the top subcell was seriously shunted. The V_OC_ and R_SH_ of each subcell were estimated using the method described before and are listed in Table 2. The results disclosed that the performance of the bottom subcell was preserved, comparable to the performance of a single c-Si cell, but the top subcell was severely shunted, which reduced its V_OC_. The damaged tandem cell behaved as if the bottom subcell was connected in series with the shunt resistance of the top subcell. The QE spectra in Fig. 8(b) also showed that the QE of the top subcell was not properly measured even though back illumination was applied, indicative of serious damage to the top subcell.

**Table 2:** The photovoltaic parameters of subcells in a partially shunted tandem cell (Fig. 8(a)), obtained from the *j-V* measurement using the cell-selective light absorption method.

Constituent Cell	V_OC_ (V)	R_SH_ (Ω)
Top subcell (CGSe)	0.451	53.8
Bottom subcell (c-Si)	0.554	22,550

## Conclusions

An electrical analysis based on the cell-selective light absorption scheme developed here successfully characterized the individual photovoltaic performances of the top and bottom subcells in a two-junction tandem cell. *j-V* measurements obtained using this scheme could estimate the values of V_OC_, R_SH_, and the bias-dependence of the top and bottom subcell photocurrents. The quantum efficiency measured using the cell-selective back light illumination enabled estimation of the respective subcell photocurrent. This method was capable of determining whether the subcells were well-functioning, shunted, or degraded during the fabrication of the c-Si/ITO/CGSe tandem cell. The results demonstrated that a monolithic tandem cell was successfully fabricated by combining a c-Si cell and CGSe cell via a specially treated ITO interlayer without electrical loss except for a slight increase in the tunnel junction resistance. This method also revealed that the performance of the tandem cell was limited by the low efficiency of the top subcell, *i.e*., a low V_OC_, low J_SC_, and a strong bias-dependent photocurrent. These key factors must be addressed to improve the performance of tandem cells.

Single cell illumination was applied to a two-junction tandem cell in this work. The method is readily applicable, with device-specific adjustments, to multi-junction tandem cells that include more than three junctions. For example, one monochromatic light source could be applied per junction such that each band is absorbed by only one junction after passage through the top subcell (with the highest band gap). A multi-junction tandem cell exposed to light absorbed by only the target subcell will provide an estimate of the open-circuit voltage of the subcell based on the x-intercept bias of the *j-V* curve, as in the two-junction tandem case. Exposure of a tandem cell to all bands other than the target subcell absorption band would provide an estimate of the shunt resistance of the subcell by analogy to the two-junction tandem case. Minimizing the intensity of the light absorbed by the target subcell while maintaining the intensity of all other illumination wavelengths could provide the bias-dependence of the photocurrent. Repeating the above procedures for each subcell could individually characterize the photovoltaic performances of each subcell.

## Methods

### Fabrication of the c-Si/CGSe monolithic tandem cell

A monolithic c-Si/ITO/CGSe tandem cell was fabricated to have the cross-sectional structure shown in Fig. 1. An aluminum back surface field (Al-BSF) cell on p-type Si was used as the bottom subcell. The emitter and Al-BSF were fabricated by first evaporating a 2-μm thick Al layer onto the back side of Si wafers, then applying a phosphorus spin-on diffusant (SOD P507, Filmtronics) onto the polished side of the wafer, and finally annealing the coated wafer at 900 °C^31^. A 50-nm-thick ITO thin film was sputter-deposited onto the surface of an n-type emitter in the c-Si cell to form a tunnel junction layer. The ITO film and emitter layer in the c-Si cell was etched out using reactive ion etching to isolate a 0.5 cm^2^ cell area. Then a 1.5-μm-thick CGSe thin film was deposited onto the patterned surface of the Si solar cell using a three-stage co-evaporation process^32,33^. At the final stage of the three-stage process, a 24-nm-thick NaF layer was additionally deposited at 450 °C for the Na doping of the CGSe films. A 60- to 70-nm-thick cadmium sulfide (CdS) layer was then deposited by chemical bath deposition^33^. An intrinsic ZnO (i-ZnO) layer of thickness 50 nm and an Al-doped ZnO (AZO) layer of thickness 500 nm were deposited by radio frequency sputtering and at the same time patterned using a metal shadow mask to define the area of the top subcell. A Ni(50 nm)/Al(800 nm) grid pattern was positioned to the patterned area of the AZO layer by ebeam evaporation. GaOx formation between the ITO and CGSe was suppressed by applying reaction-prevention treatments to the ITO layer prior to depositing the CGSe layer, which will be elaborated in a later publication.

### Electrical characterization

The current-voltage (*j-V*) characteristics of the devices were measured under air mass (AM) 1.5 illumination using an Oriel Sol3A solar simulator (Newport), and external quantum efficiency (EQE) measurements were conducted. Because the c-Si/ITO/CGSe tandem cell consisted of two subcells connected in series, accurate measurements of the EQE required that both subcells be illuminated at the same time. In other words, the absorption of monochromatic light by one subcell during the EQE measurements required simultaneous back-lighting of the second subcell at an intensity exceeding that of the monochromatic light. Without this illumination configuration, no photocurrent was generated in the second subcell, which hindered the flow of the photocurrent generated in the first subcell. Back-light illumination of the bottom subcell during the EQE measurements was applied using a 635 nm laser diode with a 4.5 mW power (Thorlabs, CPS635S), and an 850 nm laser diode with a 3.5 mW power (Thorlabs, CPS850S) was used to illuminate the top subcell. Electrical analysis of the device, described further in the next section, was conducted by applying two optical filters to the solar simulator to obtain cell-selective light absorption. A short pass filter (S86FG11) that transmitted light below 650 nm was used to generate a photocurrent only in the top subcell, and a long pass filter (830FG) that transmitted light above 830 nm was used to generate photocurrent only in the bottom subcell.

### Data availability

All data generated or analyzed during this study are included in this published article.
